# Perfusion Window Chambers Enable Interventional Analyses of Tumor Microenvironments

**DOI:** 10.1002/advs.202304886

**Published:** 2023-10-23

**Authors:** Anastasia Korolj, Rainer H. Kohler, Ella Scott, Elias A. Halabi, Kilean Lucas, Jonathan C.T. Carlson, Ralph Weissleder

**Affiliations:** ^1^ Center for Systems Biology Massachusetts General Hospital 185 Cambridge St, CPZN 5206 Boston MA 02114 USA; ^2^ Department of Systems Biology Harvard Medical School 200 Longwood Ave Boston MA 02115 USA; ^3^ Cancer Center Massachusetts General Hospital 55 Fruit Street Boston MA 02114 USA

**Keywords:** cancer, drug development, imaging, microscopy, TME

## Abstract

Intravital microscopy (IVM) allows spatial and temporal imaging of different cell types in intact live tissue microenvironments. IVM has played a critical role in understanding cancer biology, invasion, metastases, and drug development. One considerable impediment to the field is the inability to interrogate the tumor microenvironment and its communication cascades during disease progression and therapeutic interventions. Here, a new implantable perfusion window chamber (PWC) is described that allows high‐fidelity in vivo microscopy, local administration of stains and drugs, and longitudinal sampling of tumor interstitial fluid. This study shows that the new PWC design allows cyclic multiplexed imaging in vivo, imaging of drug action, and sampling of tumor‐shed materials. The PWC will be broadly useful as a novel perturbable in vivo system for deciphering biology in complex microenvironments.

## Graphical abstract

Implantable perfusion window chambers (PWC) are 3D‐printed in plastic and implanted in mice to study the tumor microenvironment dynamically. Left: color‐coded CT imaging of a mouse with dorsal PWC affixed. Right: the confocal image of the tumor microenvironment (vasculature in green; tumor cells in red; scale bar: 50 µm).

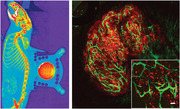

## 1 Introduction

Intravital microscopy has revolutionized the visualization and dynamic quantification of cellular and biological processes in live animals at the (sub)cellular level.^[^ ^1^, ^2^ ^]^ While certain endoscopic approaches have been translated clinically,^[^ ^3^ ^]^ most such experiments are done in mouse models through implantable window chambers that enable longitudinal imaging. A variety of dorsal (DWC) and other window chambers have been described.^[^ ^4^, ^5^, ^6^, ^7^, ^8^, ^9^, ^10^, ^11^, ^12^ ^]^ and used for major new biological discoveries^[^ ^13^, ^14^, ^15^, ^16^, ^17^, ^18^, ^19^, ^20^ ^]^ Implantable chambers are typically machined out of metal and define a static observation volume, that is, they do not allow fluid exchanges during imaging. There has long been an interest in developing perfusable window chambers (PWC) to: i) administer drugs topically, ii) rapidly compare different drug effects in the same environment, iii) perform multiplexed cyclic imaging for a deep understanding of biology, and iv) sample tumor interstitial fluid during therapeutic interventions or tumor development. This, however, has proven difficult because of: i) small sizes of parts and channels, ii) lack of rapid prototyping, iii) high cost for micromachined parts, and iv) the need for sterility.

We reasoned that it should be possible to integrate advanced microfluidics into wearable, implantable chambers and thus perform longitudinal in vivo microscopy with the in situ perturbation experiments in live mice. We further reasoned that the latest generation of 3D printers may now have sufficient resolution to fabricate models for in vivo use, similar to printed organs^[^ ^21^, ^22^ ^]^ and prostheses.^[^ ^23^, ^24^, ^25^ ^]^ To enable examination of microenvironments in vivo, we have iteratively developed and tested different PWC models, improving on design features at each stage. After many iterations, we arrived at a fully functional 3D‐printed design, which was then used to perform a diverse range of biological experiments. We were particularly interested in integrating percutaneous staining and cycling technologies to advance multiplexing abilities during imaging and in the pharmacodynamics of local drug therapy. In situ cyclic staining, if successful, would allow functional exploration of novel cellular phenotypes, particularly in the space of immunology^[^ ^13^, ^26^, ^27^ ^]^ or tracing of cellular lineages, for example, in hematopoietic development.^[^ ^28^ ^]^ Experimental techniques that can directly observe these events offer the possibility of interrogating biological interactions, developmental transformations, and downstream responses. Here, we show the versatility of the new PWC for interrogating live biology.

## 2 Results

### 2.1 Design of a Robust Perfusion Window Chamber

**Figure 1:**
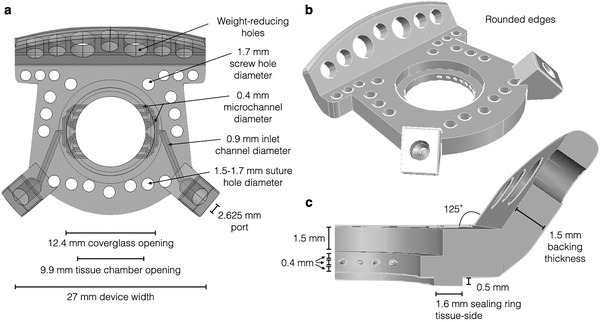
Computer‐aided design (CAD) model of the optimized perfusion window chamber (PWC). a) Semitransparent view from the top of the PWC with overall dimensions and microchannels visualized. b) Perspective view. c) Cut‐out view showing chamber dimensions and vertical spacing. The replaceable coverglass lies in a 12.4 mm diameter recess above the tissue chamber opening, with the tissue surface level immediately beneath the microchannel space.

Dorsal window chambers are well‐established model systems to visualize biological processes in live mice over time. A major caveat to these systems is the current inability to directly instill therapeutics or diagnostic agents into the chambers and to sample the tumor microenvironment (TME) while imaging in real time. Building from the design of widely used metal chambers, we identified and innovated on several key objectives and constraints (Table S1, Supporting Information) to engineer a 3D‐printable plastic device that would allow access through a chamber inlet and outlet during imaging (**Figure** **1**; Figure S1, Supporting Information).

Figure S2 (Supporting Information) summarizes some of the iterative design considerations. Most importantly, these included: i) fabrication performance with different printable materials and 3D printing technologies; ii) the microfluidic chamber design to optimize perfusion and minimize clogging, dead volumes, and pressure gradients during operation; iii) the tubing adaptors; iv) the ability to sterilize implantable chambers; and v) weight and cost factors.

Using rapid prototyping, we iterated on the design to incorporate mechanical and biological handling criteria. We required high micro‐channel resolution within a small overall thickness to maintain short working distances for microscopy while being structurally robust, stably perfusable, and able to withstand pressure drops at tubing connection points. In addition, fluid dynamics simulation (COMSOL modeling) was used to verify flow patterns between different microfluidic channel designs, to confirm uniform chamber filling, velocity, and minimal channeling of flow lines to better support device stability during operation and efficient delivery of antibody labeling agents (Figure S3, Supporting Information). The different simulated channeling properties were similarly observed in vivo (Movie S1, Supporting Information). The material also had to be biologically compatible, resistant to sterilization methods, lightweight yet durable over long implantation timescales, and reasonably priced. We evaluated stainless steel, acrylic, polystyrene, and other reinforced plastic materials printed via polyjet, fused deposition or fused filament modeling (Figure S2A, Supporting Information). Polyjet 3D printing with acrylic‐based polymer inks was most suited for reliably fabricating 400 µm‐diameter micro‐channels with architecturally correct fluid paths and a 400 µm minimum wall thickness. Below this threshold, the material would lack integrity and become brittle. Immediately after printing, the micro‐channels would inevitably be clogged by support resin, which standard washes could not clear with accepted solvents. Instead, we relied on physicochemical degradation of the support resin (Figure S2B, Supporting Information) using lyophilization in combination with higher‐order microfluidic design considerations (Figure S2C, Supporting Information). By adjusting the micro‐channel layout to have rounded corners instead of square junctions, and multiple tiers of short bifurcating channels instead of long channels on a single manifold, we were able to achieve on average 90–100% (90 ±13% mean± SD) of perfusable channels (Figure S2D, Supporting Information). Three‐tier (8‐channel) designs were more efficient in perfusion than two‐tier (4‐channel) designs. We also tested multiple connection types for their ability to stay in position when pressurized, be easily swapped in and out, remain non‐obstructive during imaging, and not degrade while implanted in vivo for weeks at a time (Figure S2E, Supporting Information). Embedded female mini‐Luer ports with adapters were most versatile.

A limited set of material options are currently available for 3D printing with enclosed channels at high resolution, and our iterations showcase the complex interplay of performance factors with material and manufacturing process choices. While polystyrene plastics are commonly used in cell culture and offer good inertness, our polystyrene‐based 3D‐printed prototypes did not demonstrate sufficient durability and channel integrity at the scales we required in the window chamber imaging system. Rubber‐like materials were also not compatible with long‐term in vivo implantation and tended to get chewed off or damaged over time despite meeting resolution requirements. While a metal PWC would be suitably inert and highly reusable, the volume required to support enclosed microchannels ultimately resulted in parts that were too heavy. Acrylic materials are biocompatible and used for implantable lenses^[^ ^29^ ^]^ or bone cement^[^ ^30^ ^]^ and offer chemical resistance to sterilizing agents. Ultimately, the acrylic‐based print material best met our needs and was thus selected for ongoing prototyping.

We initially disinfected the chambers with 30% isopropanol but observed suppuration in vivo. While antibiotics improved the inflammatory reaction, we reasoned that final design versions ultimately had to be autoclavable. We specified a dry‐heat autoclaving scheme that preserved the plastic material and rendered chambers completely sterile. These autoclaved devices were then used for subsequent biological experiments. Further to washing chambers to unclog micro‐channels, each chamber was also washed extensively with deionized water and conditioned with sterile phosphate‐buffered salie (PBS) to remove residual alkaline solvent and PWC leachate (Figure S2F, Supporting Information). The leachate from the PWC was additionally confirmed to be non‐toxic to tumor cells and fibroblasts and had negligible chemotactic effects on peripheral blood mononuclear cells (PBMCs) at low concentrations (Figure S4, Supporting Information).

**Figure 2:**
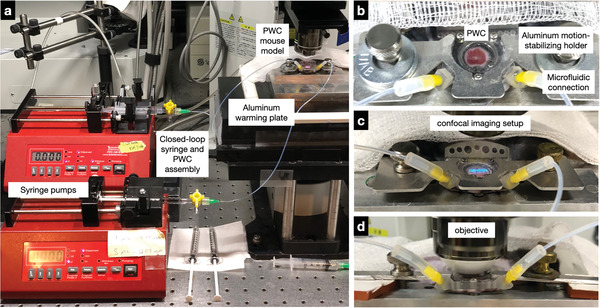
Overall imaging set‐up. a) Set‐up demonstrating dual perfusion pumps and the confocal imaging system with a mouse on a heating plate. The perfusion window chambers are connected to syringe pumps via assembled Teflon tubing. b) Image of PWC securely attached to the heating plate to avoid motion during microscopy. c,d) View of connected PWC under microscope objective during confocal imaging and perfusion.

**Figure 3:**
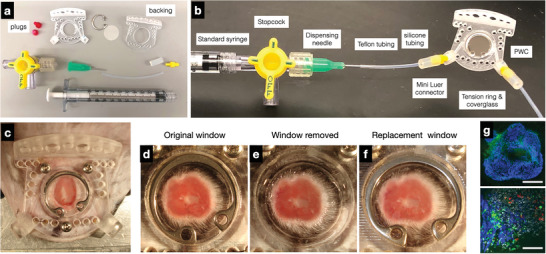
Modular components of the PWC. a) The PWC consists of a transparent, polyacrylic face plate, with backing and plugs, that accepts a glass window and perfusion tubing via mini Luer locks and microfluidic connectors. b) Window components. c) Assembled and implanted window. d–f) Once the window chamber is implanted, the glass window can be removed, cleaned, and a new window re‐inserted. This ensures access to the tumoTME and the ability to remove collagen/fibrin sheaths or debris that may have accumulated. g) Low and high magnification confocal images of the window chamber tissue from f) following replacement (13‐day‐old MC38‐TagBFP2 tumor, mertK‐GFP transgenic mouse, MHCII‐AF555, Ly6G‐AF647). AF: AlexaFluor. Upper scale bar: 2 mm. lower scale bar: 100 µm.

Beyond the above design criteria, we also optimized the experimental setup to include immobilization of the chambers on a heated warming plate to avoid hypothermia during imaging (**Figure** **2**). We initially perfused the chambers with a single syringe pump but realized that this led to tissue deformation because of pressure gradients through the microfluidic channels and tissue chamber. We therefore adopted a dual pump system with a feeding pump and a sampling pump. This resulted in lower pressure differences and no deformation of tissues within the chamber. We were also interested in the ability to replace the window chamber if the need arose. **Figure** **3** illustrates the modular assembly of the components and how the window chamber is secured within the plastic mold through a tension ring. Once the tension ring is removed, the window can be replaced with a new one without any impact on the ability to perform confocal or multiphoton imaging (Figure S5a, Supporting Information). Moreover, the devices could be effectively reused entirely by re‐sterilization and re‐implantation for many (over four) cycles (Figure S5b,c, Supporting Information).

**Figure 4:**
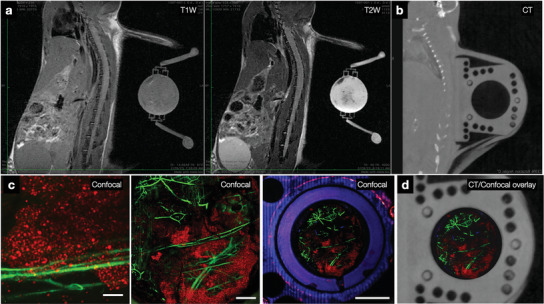
MRI compatibility of PWC. To test whether the chambers were compatible with other imaging modalities, we performed MRI and CT imaging of a live mouse implanted with a PWC. a) MRI clearly showed the TME and microchannels without any distortions. b) Similarly, micro‐CT visualized the implanted chamber. c) Confocal imaging of the same chamber after MRI imaging (tumor cells: B16‐F10 H2B‐apple in red; tumor vessels: perfused with FITC‐dextran in green). d) Both MRI and CT images could be merged with confocal images. Scale bars from left to right: 100 µm, 1, and 4 mm.

Overall, each perfusion chamber was fabricated commercially for ≈$10 (R&D Technologies). This cost is for materials only and does not include in‐house cleaning and peripheral part costs. Figure 1 summarizes the final device dimensions and blueprint, while Table S2 (Supporting Information) specifies the complete component list, and Figure S2G (Supporting Information) specifies fabrication costs of select prototypes. A final major design consideration was that the window chambers had to be magnetic resonance imaging (MRI) and computerized tomography (CT)‐compatible. The reason for this was twofold: i) to seamlessly integrate macroscopic, mesoscopic, and microscopic imaging; and ii) to make sure that implanted PWC does not interfere with MRI/CT exams done in the same animal for other reasons. **Figure** **4** shows MRI and CT images of window chambers in a live mouse, and that whole‐body imaging is not degraded by PWC. We also demonstrate the feasibility of whole‐body imaging and the fusion of macroscopic and microscopic images. We furthermore showed that both confocal and multiphoton imaging are possible through the PWC (Figure S6, Supporting Information). With intravital microscopy, signal bleed‐through from one channel to the next is always possible. To evaluate bleed‐through, we performed multichannel imaging omitting selective laser excitations. This data showed no channel bleed‐through with the current setup (Figure S7, Supporting Information).

### 2.2 Perfusion Kinetics and Cellular Labeling

We next determined the perfusion kinetics of the implanted window chambers. Figure S8 (Supporting Information) describes a key experiment in which we perfused MC38 tumor‐bearing chambers with FITC‐ and/or rhodamine‐dextran. Serial two‐color confocal imaging allowed us to quantify the perfusion kinetics and spatial distribution of the perfusate. With the fastest perfusion rate of 20 mL h^−1^, the chamber could be completely flooded and washed within 5 min. At the slowest perfusion rate of 5 mL h^−1^, a complete cycle took ≈20 min. Movie S2 (Supporting Information) shows a separate example of serial perfusion of an implanted PWC with multiple food dyes. This example demonstrates complete chamber perfusion without apparent dead spots and subsequent full washes when perfused with PBS.

We next addressed the question of whether it is preferable to stain cells in the TME via the perfusion port or by systemic administration of antibodies. To stain tumor‐associated neutrophils,^[^ ^31^ ^]^ we chose the anti‐Ly6G antibody and administered different fluorescently labeled probes systemically (2.5 µg total antibody injected in 200 µL saline; conjugated with AlexaFluor 647) or via the window chamber (4 µg mL^−1^ perfused in 800 µL PBS; conjugated with SAFE^36^‐MB488 and SAFE‐AF555). This experimental setup allowed us to determine co‐localization and signal‐to‐noise ratios of the different staining methods. As is shown in Figure S9 (Supporting Information) there was good co‐localization between systemic and chamber perfusion. The same was true when the chamber was perfused with two differently labeled antibodies. On average, the Manders I ratio was 0.95± 0.04.

A final experiment was performed to determine the depth penetration of confocal imaging with the window chamber, and different staining methods. The imaging depth by confocal imaging was ≈80 µm, similar as reported previously.^[^ ^32^ ^]^ Interestingly, there was no significant difference in Ly6G staining performed systemically or via the window chamber even at greater depths (Figure S9, Supporting Information).

### 2.3 PWC Enables Multiplexed In Vivo Imaging

**Figure 5:**
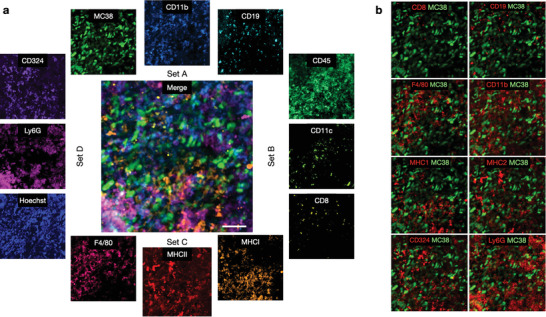
Multiplexed in vivo imaging. Immunocompetent C57BL/6J mice with a perfusion window chamber containing 7‐day‐old MC38‐TagBFP2 tumor cells (green) were imaged over four staining and cleaving cycles with different antibody conjugates. The markers included MC38‐BFP tumor cells and the other markers shown. a) Summary of the 12 channels imaged in vivo. The central merged image shows the combination of 4 channels (MC38, CD11b, MHCI, and Ly6G). Note the complementary staining possible during different in vivo cycles. Scale bar: 50 µm. b) Merged images of immune cell components (red) with the MC38 tumor cells. The raw images correspond to the ones shown in panel (a).

While the above experiments were set out to image up to four colors, as is typical for confocal and multiphoton imaging, we also wanted to test whether it would be possible to perform cyclic in vivo imaging^[^ ^33^ ^]^ as is done in spatial biology experiments in tissue sections.^[^ ^34^, ^35^ ^]^ This would drastically increase the number of cells or biomarkers that could be identified and tracked, facilitated by the ability to perfuse additional stains and quenchers through the access port. We leveraged a previously described bioorthogonal immolative technique where fluorochromes could be cleaved from a labeled antibody through highly efficient tetrazine/*trans*‐cyclooctene (Tz/TCO) scission reactions.^[^ ^36^ ^]^ In round 1, we thus labeled tumor‐associated neutrophils with anti‐Ly6G‐AF647 and obtained images. We next perfused the chamber with the Tz scissors (60 µµ HK‐Tz in PBS with 15 min incubation, followed by 3 mL PBS wash), rendering cells de‐stained, achieving a change in mean fluorescence intensity from 517 ± 88 A.U. when stained, down to 19± 9 A.U. after cleaving (*p* < 0.0001 by one‐way ANOVA). In the second round, we repeated the same experiment staining the same cells and cleaving off the fluorochrome again, this time achieving new fluorescence values of 351± 76 after stain 2 and 0 ± 8.9 after cleave 2 (*p* < 0.0001 by one‐way ANOVA). Figure S11F (Supporting Information) summarizes the signal intensity of stained and cleaved signals in cells over 2 cycles. Having shown the ability to perform cyclic imaging via the perfusion window chamber, we next set out to perform multichannel multicyclic imaging of the tumor‐immune microenvironment within the PWC. This experiment shows that it is possible to image multiple channels over at least 4 cycles, revealing 12 different targets or types of immune and cancer cells (**Figure** **5**).

### 2.4 Locally Administered Drug Action can be Imaged Through the PWC

**Figure 6:**
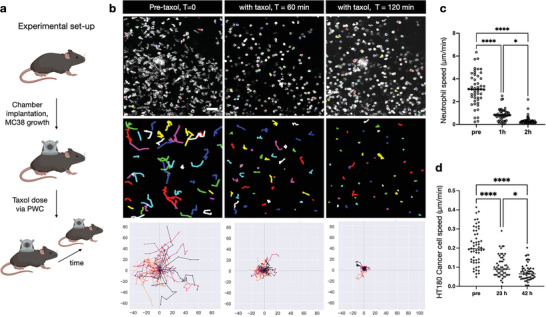
Serial in vivo imaging was performed while administering a model drug (Taxol) through the perfusion window chamber. A potentially significant application of the PWC is to serially image drug action at the single‐cell level. a) Experimental setup to image Taxol effects on immune cells and tumor cells over time. b) Quantifying cell motility (neutrophils in this example) before 60, and 120 min after perfusion with Taxol. Note the significant decrease in the motility of neutrophils with drug administration. Scale bar: 50 µm. c) Quantification of neutrophil speed, following Taxol administration. d) Quantification of tumor cell motility following Taxol administration. Compared to neutrophils, tumor cells move more slowly. Nevertheless, there is a near complete stop of motility within two days after administration. c,d) One‐way ANOVA with Tukey multiple comparisons test, *n* = 3 experiments with one representative dataset shown, mean ±SD shown, considered significant at *p* < 0.05 with *p < 0.05, **p < 0.01, ***p < 0.001, ****p < 0.0001.

A major advantage of having port access to the perfusion chamber is to add labeled and unlabeled drugs directly to the interstitial space surrounding the tumor and observe drug actions. Because of this local administration (akin to intratumorally injected drugs), the drug doses are considerably lower than for systemic administration, reducing the cost of expensive therapeutics. Drugs can also be given as a pulse‐chase experiment to test multiple drugs during one imaging session. Following the implantation of MC38‐mTagBFP2 tumors into the chamber of C57BL/6J mice, we tested how the local administration of taxol would affect immune and cancer cells. **Figure** **6**, along with Movie S3 (Supporting Information) compares the cell motility of slow‐moving tumor cells and fast‐moving tumor‐associated neutrophils. There was a significant decrease in the motility of neutrophils within 60 min of taxol administration, with a near complete stop of all cell movement by 120 min (mean neutrophil speed was 3.1 ± 1.4 µm min^−1^ at native state, 0.8+/−0.5 µm min^−1^ at 1 h after taxol, and 0.3 ± 0.5 µm min^−1^ at 2 h after taxol, with adjusted *p*‐values of *p* < 0.0001 for native versus 1 and 2 h speed, and *p* = 0.0125 for 1 versus 2 h speed). Tumor cells also showed decreased movement, however, at lower speeds and timescales. These findings are in line with the known mechanism of tubulin inhibition and subsequent cell death induced by taxol.^[^ ^37^ ^]^

**Figure 7:**
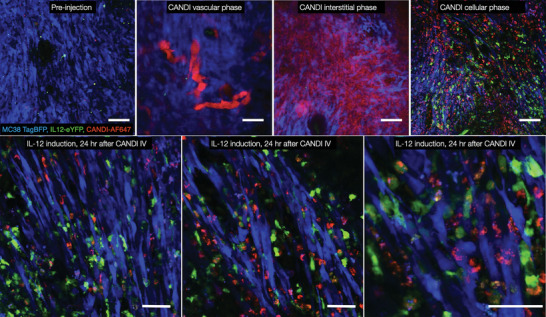
Imaging delivery drug action of a myeloid‐directed nanotherapeutic. Immunocompetent IL‐12‐eYFP mice (*n* = 5) were implanted with a PWC into which MC38‐tag BFP was seeded 8 days prior to the drug experiment. The nanotherapeutic drug (CANDI) was labeled with AF647 and contained a triple small molecule cocktail (R848, Lcl‐161, and JAK inhibitor) to induce IL‐12 in myeloid cells located in the tumor microenvironment. The top row (scale bars 100 µm) shows pre‐injection images of the tumor microenvironment; tumor cells (blue) are ubiquitously distributed and only very scant IL‐12 positive cells (green) are seen. Immediately after intravenous injection of CANDI, the nanotherapeutic drug is largely confined to vessels. Within an hour, the CANDI distributes through the interstitial space, and is later taken up by tumor‐associated macrophages in the cellular face. The lower row shows (scale bars 50 µm) high‐resolution images of the cellular distribution of CANDI to TAM and induction of IL‐12 in moving dendritic cells as well as in tumor‐associated macrophages. Note the massive IL‐12 induction within 24 h after systemic CANDI administration as well as the superb image clarity through the PWC in the live mouse.

**Figure** **7** shows an additional example of imaging the pharmacokinetics and pharmacodynamics to understand the behavior of new drugs. We have been interested in developing myeloid cell therapeutics that could be used to enhance and jumpstart a more effective immune response in tumors.^[^ ^38^, ^39^, ^40^ ^]^ In order to achieve this goal, it is frequently necessary to image different cellular targets and processes, which can be facilitated by the PWC setup. CANDI is a biocompatible succinyl‐cyclodextrin polymeric drug carrier with affinity for tumor‐associated macrophages.^[^ ^38^, ^40^ ^]^ It contains small molecule drugs such as TLR7/8 agonist (R848), cIAP inhibitor (Lcl‐161), and a JAK1 inhibitor (ruxolitinib). In vitro, the drug induces IL‐12 via the canonical and noncanonical NFkB pathways and by inhibiting IL4 signaling. The in vivo behavior, however, had yet to be ascertained. We therefore installed PWC into IL‐12‐eYFP reporter mice and seeded the windows with MC38‐mTagBFP2 tumors. At a later time point (8 d), we administered CANDI systemically and imaged drug action by conical imaging in live mice (Figure 7). Our results show that CANDI accumulated in TAM and subsequently induced IL‐12 in both TAM and dendritic cells.

### 2.5 Sampling the Tumor Microenvironment via a Closed‐Loop System

In the final proof‐of‐principle experiment, we determined whether it would be possible to sample the TME for downstream molecular analyses. We therefore implanted MC38 tumors into the PWC and sampled the TME using the PWC 7 to 10 days later. We were specifically interested to determine whether we could collect extracellular vesicles (EV) shed by tumor and other cells and detect them by in vitro analysis.^[^ ^41^ ^]^ This is important since tumor cell shedding of EV has been proposed as the major origin for circulating EV but more data is needed on local production and actual shed rates in vivo.^[^ ^42^ ^]^ With our experimental setup, we were indeed able to determine the presence of EV in the TME (Figure S12, Supporting Information) that could be effectively labeled with EV‐specific markers CD9 and CD63, suggesting the capacity for further downstream biomarker analyses and multiplexing.

## 3 Discussion

Many dynamic biological processes cannot be easily investigated by static in vitro experiments. Examples include temporal cell‐cell interactions, cellular movements, spatial heterogeneity, niche formations, reactions to chemokine and cytokine gradients, behavior of short‐lived systemically dispersed immune cell populations— all of which require longitudinal observation of cellular players. For these and related questions, intravital microscopy has become an essential investigative technology tool. However, one of its biggest impediments has been the inability to pharmacologically manipulate cellular players within chambers in near real‐time. To enable this, we have developed a 3D printed microfluidic‐containing perfusion window chamber that can be implanted into mice. Our results show that the iteratively designed chamber is cost‐effective, highly reproducible, biocompatible, sturdy, and allows interrogation of in vivo biology. While we only performed key experiments to show proof of principle, the new PWC should enable many future experiments and thus contribute to our knowledge of dynamic biology.

A number of different window chambers have previously been described.^[^ ^4^, ^5^, ^43^, ^44^ ^]^ The vast majority of these chambers were designed to be implanted in different body regions. Examples include the brain,^[^ ^45^, ^46^ ^]^ thoracic,^[^ ^47^ ^]^ dorsal,^[^ ^4^, ^7^ ^]^ and mammary chambers.^[^ ^48^, ^49^, ^50^ ^]^ These devices are mostly static, machined out of metal or optically transparent materials^[^ ^51^ ^]^ and do not allow perfusion of the underlying organ or host microenvironment. One report from our group^[^ ^52^ ^]^ previously described the possibility of 3D printing plastic chambers without microfluidic channels. In subsequent work, we added inflow and outflow by gluing needles into the chambers. Subsequent fluid mechanic modeling demonstrated uneven window chamber perfusion and local pressure gradients that were unstable experimentally. One way to circumvent these caveats is by adding microfluidic channels to distribute inflow and outflow more evenly (Figure 2).

Fortunately, advances in recent 3D printing technology have enabled high‐resolution designs to be rapidly printed and tested. Here, we show that of all the materials and printers tested, the polyjet matrix print process with acrylic‐based resins was well‐suited for prototyping chamber designs. Furthermore, with advanced physicochemical washing protocols, micro‐channels filled with support resin could be effectively cleared. While the ability of 3D printing was important for the iterative designs, we ultimately envision mass production techniques to create PWC at economically feasible scales. Several such techniques are suitable for lightweight, rigid microfluidic fabrication, the most common being micro‐injection molding combined with laser welding to generate closed micro‐channels.^[^ ^53^ ^]^ Machining closed micro‐channels has traditionally been technically challenging as feature sizes become smaller, but recent advances have opened up new possibilities.

A key question with the implantable perfusion chamber was whether there were inflammatory responses and whether these would be more pronounced in those observed with traditional metal window chambers. In early implanted prototypes, we indeed saw an inflammatory response, primarily neutrophil accumulation in the chamber. We hypothesized that this neutrophil response could be due to i) sterility issues, ii) the leaching of chemicals from the plastic, and iii) a foreign body response to the implantation. We subsequently performed optimization experiments that led to sterilization and extensive washing protocols of the PWC before implantation. With this improved protocol, the inflammatory response of PWC is virtually identical to the well‐accepted metal window chambers, supporting the addition of PWC as a reliable surgical model system for intravital microscopy. Moreover, the lightweight plastic PWC with rounded edges often outperformed metal chambers while implanted, as the smoother edges were less prone to cutting the skin than the thin metal chambers.

Microfluidics have also been used extensively for cell culture, organoid, organ‐on‐a‐chip systems, and in vitro staining of co‐cultures.^[^ ^54^, ^55^, ^56^, ^57^, ^58^ ^]^ For example, microfluidic platforms enable the controlled addition of fluid flow and mechanical stress to cells grown in vitro.^[^ ^59^, ^60^, ^61^, ^62^ ^]^ These dynamic culture environments promote investigating mechanosensitive cellular processes such as proliferation, alignment, and remodeling. However, the dynamic culture systems are often far from physiological reality, where many other biological factors and cells are at play. Thus, to our knowledge, the PWC developed here is the first device incorporating dynamic cultures with in vivo observations.

To disseminate the technology, we provide CAD drawings of our optimized window chamber so others can reproduce it. We anticipate future improvements and additional biological experiments as others use this emerging technology. During the development of the current PWC, we overcame a number of design hurdles, which resulted SIin a well‐working system (Table S1, Supporting Information). However, some potential limitations remain. Perhaps the biggest limitation of the current design is the presence of a larger gap that needs to be filled by growing tumors, which can take longer than with thinner metal chambers as tissue is not pressed up against a glass window. Future enhancements could include the emergence of new biocompatible plastics with improved physical properties to allow for thinner materials, better printing technology to enable smaller channels, or designs micro‐machined out of titanium or other biocompatible metals. Designs may also be adapted for other organ imaging applications. Another potential limitation is the occasional clogging of channels, which can be unclogged with warm solutions. Future material enhancements may allow thinner chambers and wider channels to further avoid this rare occurrence. Future scaling and mass production through different molding technologies will likely overcome these minor limitations and result in systems efficiently mass‐produced at affordable costs and high quality.

## 4 Experimental Section

### Device Fabrication

PWC models were drafted in AutoCAD for Mac as solid parts with the 3D modeling interface. Versions were exported from .dwg to .stl files and sent to R&D Technologies Inc. for 3D printing on a Stratasys PolyJet Matrix J850, using proprietary VeroClear RGD810 acrylic‐based photopolymer as the build material and Sup706B as the support material. Other technologies and materials tested included fused deposition modeling (FDM) of ABS‐M30i on a Fortus 450mc with R&D Technologies Inc., and fused filament modeling (FFM) of 17‐4 PH stainless steel on a Metal X system with MarkForged Inc. Modeling and prototyping was iterated in batches, adjusting the variables in Table S1 (Supporting Information) until an optimized window chamber was developed.

### Computational Fluid Dynamics Modeling

PWC designs were modeled using COMSOL Multiphysics Computational Fluid Dynamics modeling software (COMSOL). PWC models were imported to COMSOL as DXF files and modeled using the fluid flow physics package for steady state flow. Inlet velocity was calculated as a function of the inlet flow rate used experimentally and the cross‐sectional area of the flow inlet and outlet velocity was set to atmospheric pressure. Devices were modeled to scale to ensure accurate representation of the system. Velocity and pressure scale bars of all models were scaled to the maximum velocity and pressure of the chosen design for accurate comparison.

### Device Washing, Sterilizing, and Reuse

To remove and unclog residual support material from channels and prepare parts for in vivo use, parts were washed in a room‐temperature sealed baths with agitation using 10 mL solution per part. The sequence was as follows: (day 1) Parts were washed in 1% sodium hydroxide solution in deionized (DI) water overnight, followed by; (day 2) three 5 min washes in DI water and an overnight wash in DI water; (day 3) parts were transferred from the bath into a dry container and placed in −30 ˚C overnight; (day 4) parts were transferred from the freezer to a lyophilizing jar and freeze‐dried for 24 hr; (day 5) parts were rinsed with three 5 min agitated washes in DI water, checked for perfusion by injecting DI water with blue food coloring at the inlet ports with a fitted syringe, rinsed to remove the color, then washed for an additional 1–7 days in DI water, replenished daily. Longer washing at this stage was preferred for reducing suppuration around the PWC in vivo. After washing, parts were further soaked for 30 min in 30% isopropyl alcohol solution in DI water, followed by one 5 min rinse with DI water and an overnight wash in Dulbecco's phosphate buffered saline (DPBS). Parts were dried with compressed air before packaging for autoclaving. The pH of supernatant water was measured at several time points throughout the wash protocol to monitor alkalinity reduction, for *n* = 3 separately washed parts.

For part reuse, used chambers were removed surgically and manually rinsed with water, followed by agitated washing with water, checking channel perfusion, then autoclaving. Soaking in isopropyl alcohol was less effective than autoclaving for sterilizing, as the parts were sensitive to extended contact with isopropyl alcohol as a solvent. An accelerated wash protocol was developed to mimic multiple reuse cycles where parts were soaked in PBS in a 37 ˚C incubator, autoclaved, then checked for perfusion and part integrity. Parts were thus repeatedly washed until the breakdown of chamber integrity.

### PWC Leachate Cytotoxicity Assay

Washed and sterile parts were incubated for 3 days at 37 ˚C in DMEM culture media with 10% FBS and 1% pen‐strep, using 10 mL per part, to generate conditioned media to test the cytotoxicity of potential PWC leachate. The conditioned media approach was selected to mimic the exposure of cells to the PWC. Unconditioned control media was also generated. Cells from a KPT tumor cell line (WH6042 KPT, Kras^LSL‐G12D^ Trp53^Fl/Fl^ Rosa26^LSL‐tdTomato^, a kind gift from William Hwang) and from an IMR90 fibroblast cell line (ATCC, CCL‐186) were tested to represent the sensitivity of both tumor and stromal cell types to PWC leachate. The LDH assay (Invitrogen, C20300) was used according to the manufacturer's protocols to assess cytotoxicity. Briefly, cells were plated in 96‐well plates at a seeding density of 100 000 cells cm^−2^. Cells were treated the next day with 0%, 10%, 50%, and 100% conditioned media mixed with control media. The supernatant was collected from cells on day 1 and day 3 post‐treatment alongside control groups and mixed with LDH assay reagents. The positive control was a preset kit reagent. Absorbance was measured on a plate reader and normalized to background signal and control wells to determine % cytotoxicity per manufacturer's protocol.

### PWC Leachate Chemotaxis (Boyden) Assay

Mouse PBMCs were obtained via terminal collection, kept on ice, and diluted 1:1 with cold DPBS. Ficoll density gradient (Sigma–Aldrich Histopaque 1083, suitable for mouse blood) was used in a 3:4 ratio with diluted blood and centrifuged for 40 min at 400 g with brake‐off to separate PBMC from plasma and platelets. PBMC were washed with DPBS, counted, then resuspended at 100 000 cells mL^−1^ in DMEM culture media with 10% FBS and 1% pen‐strep. The cell suspension was loaded onto 5 µm‐pore transwell inserts of a 24‐well plate, whose bottom wells were pre‐filled with control or conditioned media. Cultures were incubated for 4 h, then rinsed with DPBS and the membranes fixed in 4% PFA for 20 min at room temperature. After a further three DPBS rinses, q‐tips with a flat top were used to wipe off non‐migrated cells from the top side of the transwell membranes, thereby keeping only migrated cells on the membrane bottom side. Scalpel and tweezers were used to remove membranes from transwell inserts, then mounted with DAPI‐containing antifade mounting medium (3 µL) (Vector laboratories H‐2000‐2) onto microscope slides and coverglass. Mouse PBMC migration across 5 µm‐pore transwell membranes was imaged at 10× magnification, *n* = 2–5 wells per group with three sample images per well. Cells were quantified using FIJI, data points normalized to an average of 0% group, then analyzed in Prism 10 with checks for normal distribution, any outliers removed, and statistical testing with student's *t*‐test, *p* < 0.05, with the mean ±SD shown in graphs.

### Perfusion Setup

Before intravital imaging, the coverglass and tension ring were sealed with cyanoacrylate glue to prevent liquid leakage during perfusion. The rim of the glue was later peeled off after imaging. Channels and ports were filled with PBS before adding plugs to maintain a liquid seal and lack of bubbles. For a typical perfusion experiment, two segments of medical grade Teflon tubing (gauge 22) were prepared by cutting into 30 cm‐long pieces using a tube cutter. One end was directly fitted onto a Luer lock connection dispensing needle (1/2″ length and gauge 23), which was locked onto a three‐way stopcock with a pre‐filled syringe. A stopcock was used to facilitate exchanging syringes while maintaining a closed‐loop system with constant pressure and preventing the introduction of bubbles. The other end of the tubing was fitted onto a male mini‐Luer connector using a 1 cm piece of silicone support tubing. Two Teflon tubing constructs were assembled, one for the inflow pump and the other for the outflow pump. Both tubing constructs were flushed and primed with PBS before connecting to the PWC to ensure fluid continuity and lack of bubbles and to minimize pressure drops when perfusion was initialized. Typically, a 1 mL syringe was used for the inflow and 10 mL for the outflow, with the syringe barrel diameter adjusted accordingly in the syringe pump settings. Any syringe size may be swapped in via the stopcock. Care was always taken to maintain liquid contact when making any new connections to preserve fluid continuity. Syringes were initially hooked up to the PWC and perfused with PBS to stabilize the system and verify the absence of leaks before focusing the objective for imaging. A flow rate of 10 mL h^−1^ (range 5–20 mL h^−1^) was used for routine experiments, and practical solutions were loaded in syringes with ≈10% excess volume to account for dead space. Both pumps were typically started at the same time when running perfusion.

### Intravital Microscopic Imaging

All confocal images were collected using a customized Olympus FV1000 confocal microscope (Olympus America). A 2 x (XLFluor, NA 0.14), a 4x (UPlanSApo, NA 0.16), and an XLUMPlanFL N 20x (NA 1.0) water immersion objective were used for imaging (Olympus America). Tumor cells (Table S4, Supporting Information), host cells (Table S5, Supporting Information), antibodies, and vascular probes (Table S3, Supporting Information) were excited sequentially using a 405, a 473, a 559, and a 633 nm diode laser, in combination with a DM‐405/488/559/635 nm dichroic beam splitter. Emitted light was further separated by beam splitters (SDM‐473, SDM‐560, and SDM‐640) and emission filters BA430‐455, BA490‐540, BA575‐620, and BA655‐755 (Olympus America). Confocal laser power settings were carefully optimized to avoid photobleaching, phototoxicity, or tissue damage. Fiji (ImageJ, 2.9.0/1.53t) was used for image analysis.

### Cell Culture and Tumor Implantation

Cells (Table S4, Supporting Information) were subcultured at 37 ˚C and 5% CO_2_ in T75 flasks using DMEM basal media with 10% FBS and 1% pen‐strep unless otherwise specified. 0.05% trypsin‐EDTA was used for passaging. Cell lines (WH6042 KPT and IMR90) were used for cytotoxicity studies, and fluorescent tumor cells lines (e.g., MC38‐H2B‐mApple, HT‐1080‐mApple, B16‐F10‐H2B‐mApple) were used for in vivo implantation to the window chambers. Tumors were implanted in the window chambers as previously described^[^ ^5^, ^63^ ^]^ and allowed to grow for 7–21 days before imaging experiments, with tumor growth monitored regularly.

### Mouse Models

All animals were bred and housed under specific pathogen‐free conditions at the Massachusetts General Hospital. Experiments were approved by the MGH Institutional Animal Care and Use Committee (IACUC) and were performed by MGH IACUC regulations. Table S5 (Supporting Information) summarizes the mouse experiments. C57BL/6J mice or IL‐12‐eYFP reporter mice were utilized for tumor implantations.

### MRI Imaging

MRI was performed with a 4.7‐Tesla horizontal bore Pharmascan system (Bruker). Imaging parameters were as follows: T1W: TR 873 ms, TE: 13 ms; T2W: TR 4000 ms, TE: 40 ms; slice thickness: 0.6 mm; NEX 2–4. Anatomical and functional parameters were quantified using Miele software (Mac).

### Micro‐CT Imaging

CT imaging was done using a Siemens Inveon PET‐CT system. The CT uses CCD technology that allowed the highest available signal‐to‐noise ratio and fiber optics that permit the highest light collection efficiency. It had 4064 × 4064 detectors, a FOV >10 × 10 cm, a spatial resolution of 15‐micron isotropic voxels, and could scan an entire mouse in <1 min.

### FITC‐Dextran Perfusion Kinetics Imaging

A 1 mL syringe was loaded with 40 µg mL^−1^ solution (1.1 mL) of fluorescein isothiocyanate (FITC)‐dextran or rhodamine‐dextran (both with molecular weight (MW) = 2000 000 Da) in PBS. The objective was set in focus, and a time‐lapse was started 3–4 frames before starting the pumps for perfusion. The frame number was recorded at regular intervals of elapsed volume: at 400, 800, and 900 µL of fluorescent solution dispensed; when pumping was stopped for syringe exchange with PBS; and further dispensed volumes of PBS, for a total of 2500 µL perfused solution for one run of kinetic imaging. Frame number was cross‐referenced with dispensed volume to normalize graphs. 5, 10, 15, and 20 mL h^−1^ flow rates were tested on 4‐channel and 8‐channel PWCs.

### Antibody Labeling with Bioorthogonally Cleavable SAFE Fluorophores

Unconjugated purified mouse antibodies were purchased from trusted vendors, as summarized in Table S3 (Supporting Information), and selected based on prior in‐house validation. To conjugate antibodies with SAFE probes,^[^ ^36^ ^]^ a 0.1 µ sodium bicarbonate solution in PBS was prepared, and the pH was adjusted to 8.4 using 1 N hydrochloric acid. MWCO Zeba columns (40k) (Thermo Fisher, 87 765) were brought to room temperature, then equilibrated and buffer‐exchanged with the sodium bicarbonate solution. Unconjugated antibody (10 µL), typically at concentrations of 0.5–1 mg mL^−1^, was passed through the Zeba column to exchange the storage buffer. Recovered antibody concentrations were measured on a NanoDrop using the Protein280 + IgG module and volume double‐checked by reverse pipetting. Reaction volumes were prepared using an 8x molar excess of SAFE fluorophore per antibody, and the DMSO volume was maintained at 10% of the total reaction mixture. Reactions were mixed and incubated at room temperature for ≈30 min, then loaded and eluted from Zeba columns loaded with PBS to remove the free dye. The labeling conversion was measured on a NanoDrop using the UV–vis module and checking the absorbance of protein and fluorophore. Typically, antibodies with a degree of labeling (DOL) of 2–5 were used in experiments. Labeled antibodies were stored at 4 ˚C and used the next day or within a month.

### In Vivo Cycling

Cycling of in vivo immunofluorescence labeling was conducted on C57BL/6J mice implanted with MC38‐H2B‐GFP tumors. To slow down antibody internalization by macrophages and immune cells, 100 µg of anti‐FCR antibody was injected intravenously in saline (200 µL) before imaging as a blocking agent. Similarly, 2.5 µg mL^−1^ anti‐FCR was dissolved in PBS (800 µL) staining solution with 2–4 µg mL^−1^ SAFE‐labeled antibodies for a given cycle, prepared as in Table S3 (Supporting Information). Staining solution was initially perfused through the window chamber (700 µL), incubated (2 min), then rinsed with PBS (1 mL) before imaging. After a z‐stack was acquired, 60 µµ aminoethyl‐Tz solution (HK‐Tz) in PBS (700 µL) was perfused and incubated (15 min) to conduct the cleaving of SAFE fluorophores from the labeled antibodies. The cleaved system was rinsed with PBS (3 mL) to remove unbound fluorophore from the chamber. Image settings were kept constant, and a post‐cleaving z‐stack was acquired before running the next cycle of labeling and cleaving. Perfusion was typically conducted by adding solution (20‐40 µL) up‐front before the withdrawal was initiated, then withdrawing solution (20‐30 µL) on the back‐end after perfusion was stopped to adjust the tissue height to improve staining coverage and image focusing, respectively. Flo10 ml hr‐1 flowrates were used. Validation experiments used 2.5 µg primary anti‐mouse Ly6G‐AlexaFluor‐647 delivered intravenously the day before imaging (5 µL of a 0.5 mg mL^−1^ stock solution dissolved in 200 µL PBS), and 4 µg mL^−1^ SAFE‐labeled antibody dissolved in PBS (800 µL) by PWC in situ.

### Drug Testing with Taxol

For taxol experiments, C57BL/6J mice were implanted with PWC and MC38‐mTagBFP tumors. Chambers were first initialized with PBS, then perfused with FITC‐dextran to validate full chamber coverage by perfused medium then rinsed with PBS. 4 µg mL^−1^ of anti‐mouse Ly6G antibody conjugated with AlexaFluor 647 was prepared in PBS (800 µL). Staining solution (700 µL) was then perfused into the chamber, incubated (2 min), then rinsed with PBS (1 mL) to label neutrophils for assessing cell motility. A pre‐taxol movie was acquired as a z‐stack time‐lapse for 1 h. Taxol (paclitaxel, Selleck Chem, Cat # S1150) stock solution was prepared in dimethyl sulfoxide (DMSO) at 20 mg mL^−1^ and kept frozen in aliquots at −30 ˚C until use. Taxol was further dissolved to 10 µg mL^−1^ in PBS for experiments. Taxol solution (700 µL) was perfused through the PWC and left to incubate (30 min) before rinsing with PBS (1 mL). This concentration corresponded to tissue concentrations achieved with a single bolus of 30 mg kg^−1^ paclitaxel and was used based on published data.^[^ ^37^ ^]^ A 2 h z‐stack is a time‐lapse acquired after rinsing to monitor neutrophil and tumor cell motility post‐treatment. Taxol solution was kept sealed in the syringes and tubing as a closed system for the duration of the experiment.

### CANDI Experiments

The synthesis of cyclodextrin nanoparticles (CANDI) was further developed from a previously reported method.^[^ ^38^, ^40^ ^]^ Fluorescent CANDI was prepared by attaching AF647 succinimidyl ester (ThermoFisher, 2 mg mL^−1^ in DMSO). The labeled nanoparticle was purified by buffer exchange into water against 10 kDa MWCO centrifugal filters (Amicon; 10 000 rcf for 5 min; 300 µL water per wash, 3–4×). A solution of empty CANDI (5 mg) in PBS (0.1x, 90 µL) was also used for loading a mixture of payloads: ruxolitinib (0.20 mg), LCL‐161 (0.25 mg), and R848 (0.1 mg), in DMSO (10 µL). The solution was vortexed rapidly until the complete dissolution of the drugs. All solutions were filtered through a 0.22 µm sterile filter (VWR) and used immediately for characterization, in vitro assays, or stored at −20 °C until further use. The compounds were used for systemic administration to IL‐12‐eYFP reporter mice (*n* = 5) bearing a PWC, which had been seeded with 4 million MC38‐mTAGBFP2 cells 8 days prior. Confocal imaging of live mice was performed before administration, during administration for several hours, and 24 h after administration.

### Effluent Collection and EV Isolation

To collect effluent from the window chamber, the PWC was connected with a 10 mL PBS inflow syringe and a 5 mL PBS‐primed outflow syringe using 45 cm length tubing. The chamber was connected directly to tubing and pumps after 2–7 days without rinsing or opening the chamber. PBS was perfused through the chamber to collect window chamber effluent medium (3 mL). The PWC was then disconnected, tubing further drained into the syringe, and stored at 4 ˚C immediately post‐sampling. The same day, samples were transferred to falcon tubes and centrifuged at 300 g to remove cell debris. The supernatant was collected and further centrifuged at 4000 g, followed by another supernatant transfer through a 0.2 mm polyvinylidene difluoride (PVDF) syringe filter into 10 k and 4 mL volume Amicon Ultra (Millipore‐Sigma, UFC801024) sample concentrating columns. These were centrifuged at 4000 g for 15 min per the manufacturer's protocol. Concentrated samples were collected, and protein content was measured with a Qubit protein quantification system. To isolate extracellular vesicles (EVs) from the samples, the concentrated sample (100 µL) was processed through ExoSpin Mini size exclusion chromatography columns (Cell Guidance Systems, EX03‐25). The protein content of the freshly purified EV fraction was measured again on the Qubit system. To assess EV content in the PWC effluent samples, single EV imaging techniques were used. Briefly, EVs from the chamber effluent were collected and labeled with the pan‐EV tetrafluorophenyl (TFP) ester label. Due to the low expected concentration, EVs were isolated using membrane filtration and then labeled for CD9 and CD63 in situ. Extracellular vesicles captured and labeled on the membrane were then imaged as previously described.^[^ ^41^ ^]^

### Statistical Analysis

All statistical data analyses were performed using GraphPad Prism 9 software, and results are expressed as mean ± standard deviation. Data was checked for normality and for removal of outliers. And two‐tailed Student's *t*‐tests and one‐way ANOVA tests were followed by Tukey's or Fisher's LSD multiple comparison tests for normally distributed datasets. Non‐parametric Mann–Whitney or Kruskal–Wallis tests were performed when variables were not normally distributed. *p* values > 0.05 were considered insignificant (n.s.), and p values < 0.05 were considered significant. ^∗^ *p* value < 0.05, ∗∗*p* value < 0.01, ∗∗∗*p* value < 0.001, ∗∗∗∗*p* value < 0.0001.

## Conflict of Interest

R.W. is a consultant to ModeRNA, Boston Scientific, Lumicell, Seer Biosciences, Earli, and Accure Health, none of whom contributed to this research. R.W. and J.C.T.C. are co‐inventors on several patents, all of which have been assigned to MGH and are managed through institutional oversight. A.K. is an inventor on patents for unrelated work. The other authors declare no conflict of interest.

## Author Contributions

R.W. performed conceptualization. A.K. and R.H.K. performed data acquisition. E.A.H., J.C.T.C., and A.K. performed chemistry. All authors performed formal data analysis. R.W. and R.H.K. performed validation. R.W. performed supervision. R.W. performed visualization. R.W. wrote original draft. R.W. and all coauthors wrote reviewed and edited. R.W. performed funding acquisition, project administration, and acquired resources.

## Supporting information

Supporting Information

Supplemental Movie 1

Supplemental Movie 2

Supplemental Movie 3

## Data Availability

The data that support the findings of this study are available from the corresponding author upon reasonable request.
