# Supplementary material for: Perfusion Window Chambers Enable Interventional Analyses of Tumor Microenvironments
Source: Adv Sci (Weinh). 2023 Oct 23;10(34):2304886. doi: 10.1002/advs.202304886 (PMC10700240; doi:10.1002/advs.202304886)
Supplement: Supplementary file 1 — Supporting Information [file ADVS-10-2304886-s003.pdf]

ADVANCED SCIENCE
Open Access

Supporting Information

for *Adv. Sci.*, DOI 10.1002/advs.202304886

Perfusion Window Chambers Enable Interventional Analyses of Tumor Microenvironments

*Anastasia Korolj, Rainer H. Kohler, Ella Scott, Elias A. Halabi, Kilean Lucas, Jonathan C.T. Carlson and Ralph Weissleder**

Supporting information

Perfusion window chambers enable temporal analyses of tumor microenvironments.

Anastasia Korolj[1,3], Rainer Kohler[1], Ella Scott[1], Elias A. Halabi[1], Kilean Lucas[1], Jonathan C.T. Carlson[1,2], Ralph Weissleder[1,2,3]*

[1] Center for Systems Biology, Massachusetts General Hospital, 185 Cambridge St, CPZN 5206, Boston, MA 02114,

[2] Cancer Center, Massachusetts General Hospital, 55 Fruit Street, Boston, MA 02114

[3] Department of Systems Biology, Harvard Medical School, 200 Longwood Ave, Boston, MA 02115

*R. Weissleder, MD, PhD
Center for Systems Biology
Massachusetts General Hospital
185 Cambridge St, CPZN 5206
Boston, MA, 02114
617-726-8226
rweissleder@mgh.harvard.edu

Keywords: imaging, microscopy, cancer, TME, drug development

Fig. S1: 3D-printed microfluidic perfusable window chamber (PWC) enables fluid flow through mouse dorsal skin-fold window chamber for staining, drug testing, and TME sampling. a, Standard static titanium dorsal skin-fold window chamber. **b,** CAD model semitransparent and 2D outline views of the PWC and its flow path. **c,** Images of 8-channel PWC with blue dye perfused through microchannels and the assembled final version. **d,** Image of PWC installed *in vivo* with the corresponding multichannel confocal image obtained from it. Scale bar: 50 µm.

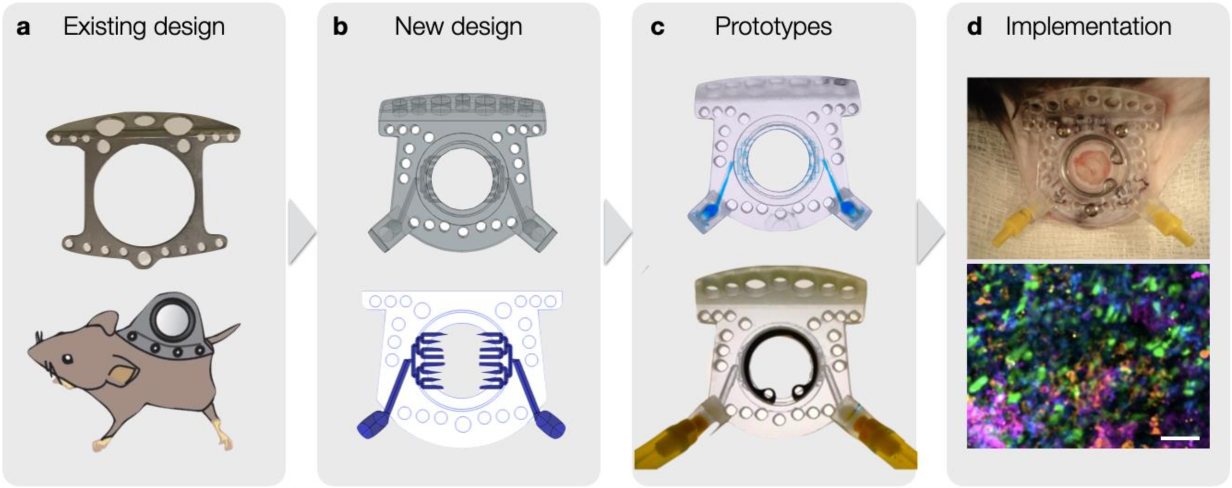

Fig. S2: Design of PWC. a, Sample of materials tested. Polyjet matrix acrylic was most reliable, perfusable, and fully autoclavable. **b,** Microchannel appearance before and after wash protocol, shown when dry and when perfused with blue dye. **c,** Samples of manifold types tested. **d,** Percentage of channels perfusable pre- and post-cleaning. n=3 devices, two-way ANOVA with Fisher's LSD test, * p<0.05, ** p<0.01, *** p<0.001. **e,** Sample of connection types tested. **f,** pH of supernatant through cleaning protocol (n=3 parts, mean ±SD). **g,** Weight and cost (as quoted by suppliers at time of writing) of standard window chamber and of PWCs made of various materials. The chambers were sterilized in isopropanol and autoclaved before implantation. Scale bars: 5 mm. PS = polystyrene, SS = stainless steel, standard = standard titanium window chamber (non-perfusable).

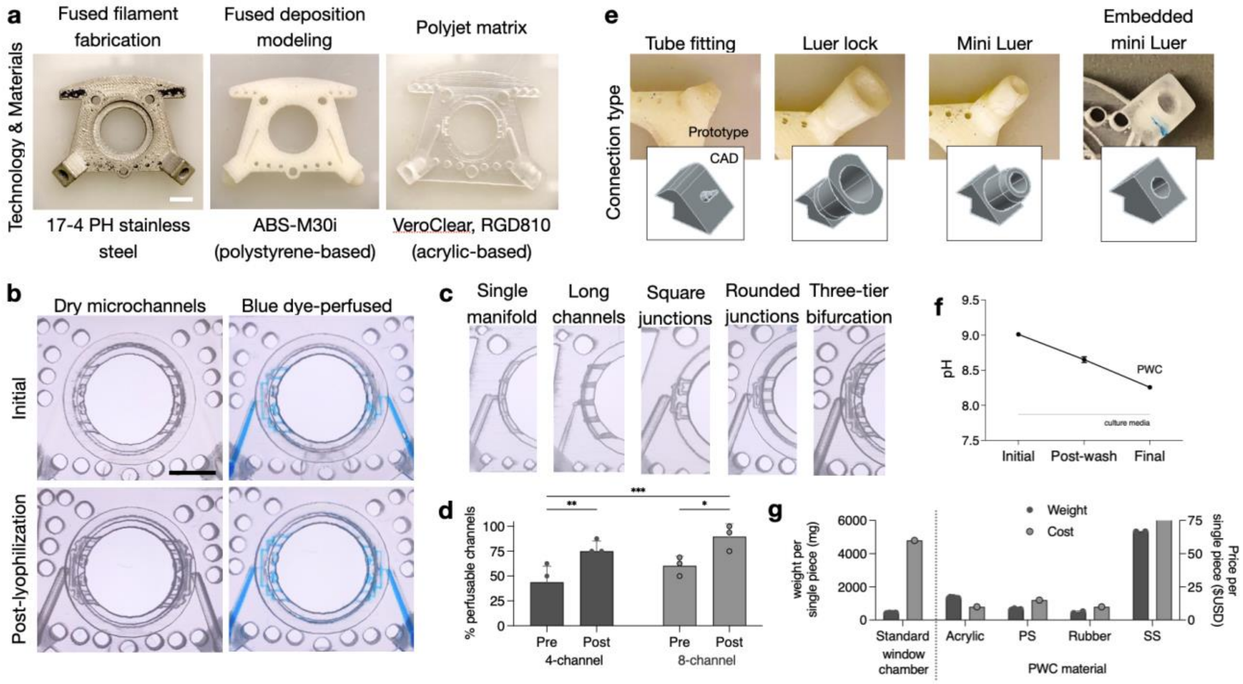

Fig. S3. COMSOL modeling of different channel designs. The overall objective of the PWC was to achieve uniform and robust filling through the chamber and absence of high/low pressure regions that could negatively affect tissue perfusion or chamber seal. Four microfluidic channel designs were simulated: **a,** 3-tier bifurcation, **b,** 2-tier bifurcation, **c,** single bifurcation with short channels, **d,** single bifurcation with long channels. The 3-tier design showed uniform velocity with minimal channeling of flow lines to support effective staining, and a gradual pressure drop between the inlet and chamber space that was contained within the device. These properties helped to maintain device stability during operation. Velocity and pressure legends are scaled to device in (a), with min and max velocities of 0.1-2 mm s^{-1} respectively at 10 mL hr^{-1} flow rate, and min and max pressures of 99-105 kPa.

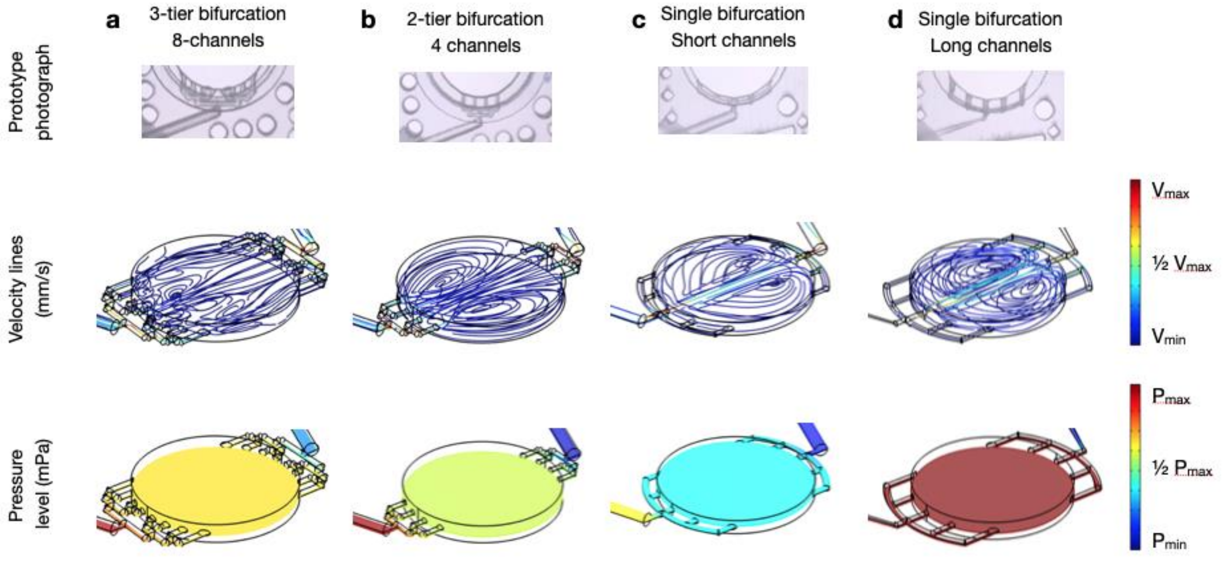

Fig. S4: PWC leachate is non-toxic and non-chemotactic in vitro. Culture media was incubated for three days with PWCs to generate conditioned media. 0%, 10%, 50%, and 100% conditioned media was used in cell culture with **a,** IMR90 fibroblast cells, and **b,** KPT tumor cells, to test for cytotoxicity of PWC leachate using the LDH assay. Cells were exposed to conditioned media for 3 days in vitro. Note that the leachate from PWC is not toxic to fibroblasts or tumor cells. n=3 replicates, with data normalized to average of 0% group, one-way ANOVA with Tukey multiple comparisons test, $p<0.05$, and mean ±SD shown. **c,** Boyden migration assay was used with freshly isolated mouse PBMCs to assess chemotactic effects when using 10% conditioned media as an approximation of chamber leachate levels. n=3 replicates repeated twice, with data normalized to average of 0% group, student's t-test, $p<0.05$, and mean ±SD shown. LDH: lactate dehydrogenase; PBMC: peripheral blood mononuclear cells.

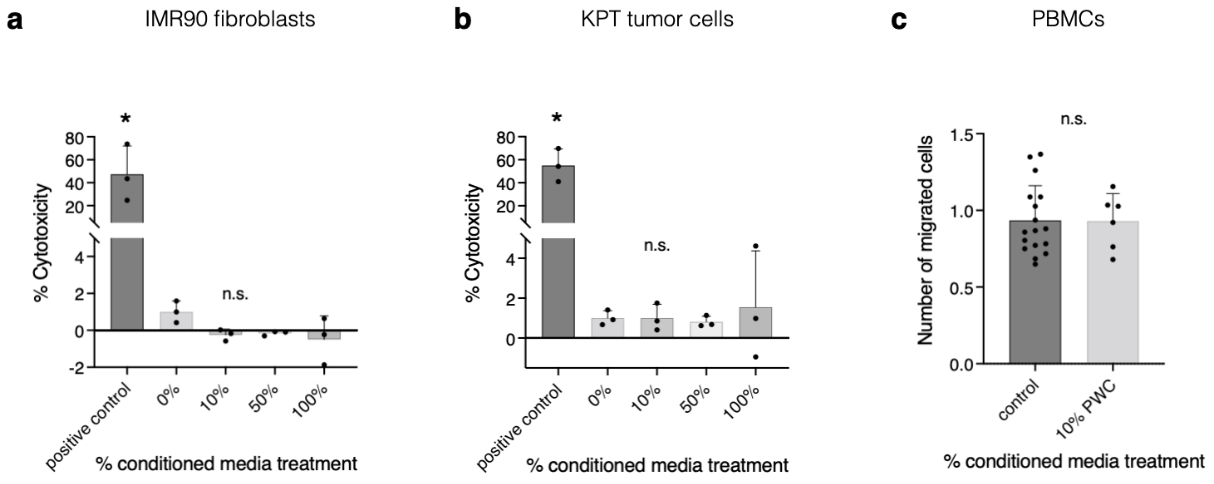

Fig. S5: Re-usability of PWC. a, Three different PWC (chambers F, K, and L) were implanted into other mice, imaged, explanted, cleaned, re-sterilized, and then re-implanted. For example, chamber F was implanted into mouse 1, then mouse 4, then mouse 7, then mouse 10. MC38-H2B-apple cells were implanted in mice 2 and 3 to allow comparison between channels. Note that the PWC function properly over time, can be repeatedly re-sterilized, and image quality does not degrade. **b-c,** Parts were also serially washed in a 37˚C bath and autoclaved to monitor part integrity over time. b, Before and after images of PWC and backings after autoclaving. **c,** Number of PWC and backings that remain perfusable (PWC) and intact (backing) with full integrity after each autoclaving cycle.

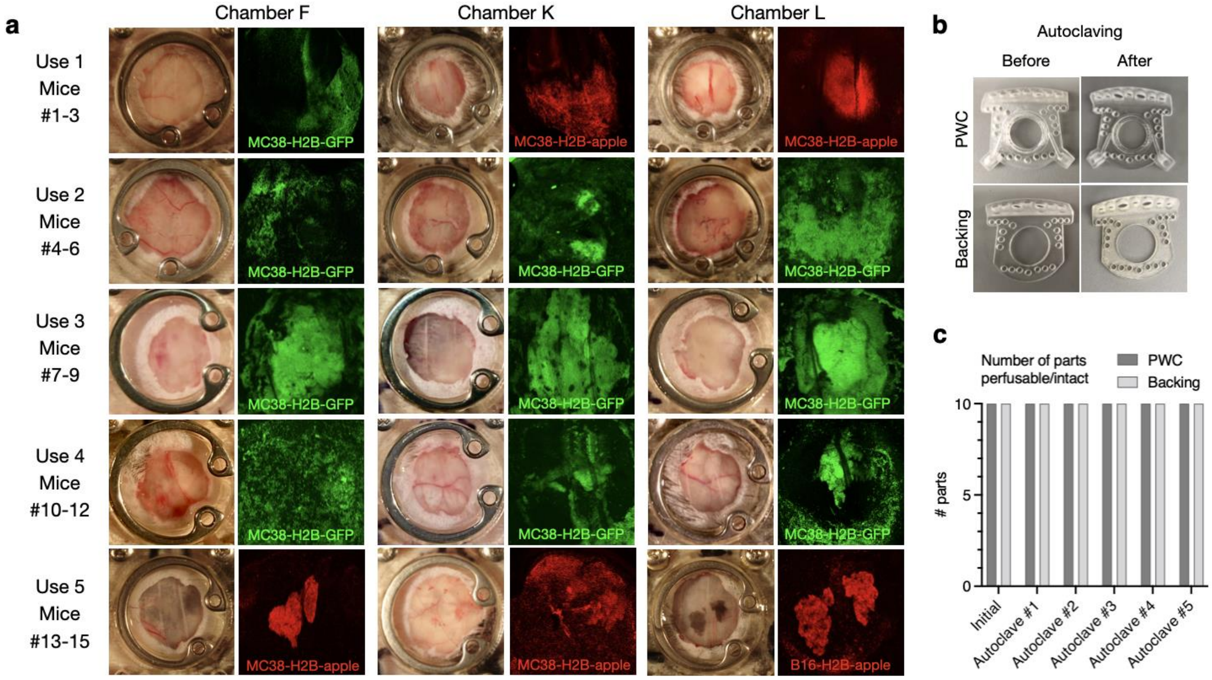

Fig. S6: The PWC allows both confocal and multiphoton imaging. B16-F10-H2B-mApple or MC38-TagBFP2 fluorescent tumor cells were implanted into PWCs on day zero. 7 to 10 days after tumor injection, mice (C57BL/6J or mertK-GFP) were imaged using intravenous administration of fluorescent dextrans or GSL I (lectin) as a vascular label. Both **a,** confocal and **b,** multiphoton imaging were performed through the window chamber in live animals for both tumor models. Note the excellent correlation between the two imaging modalities. Scale bars: 100 µm.

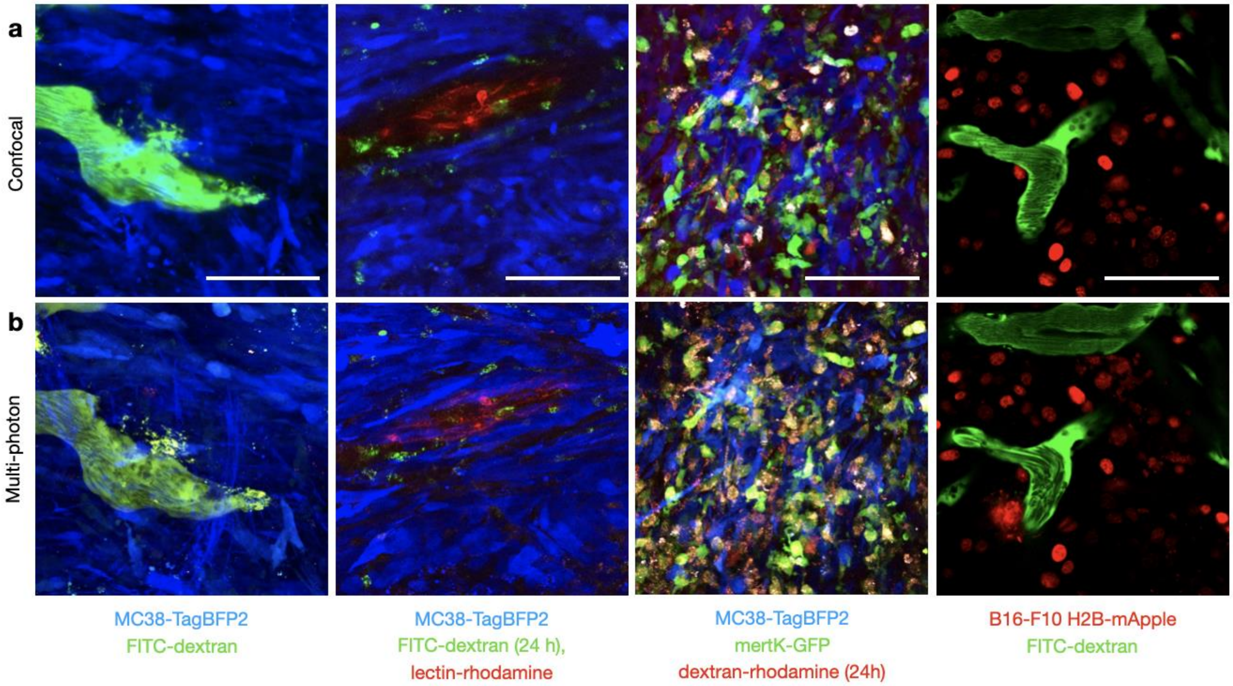

Fig. S7: Selective image acquisition through PWC without bleed-through. MC38-TagBFP2 fluorescent tumor cells were implanted into six C57BL/6J mice and imaged 7-10 days later. Mice received aliquots of anti-Ly6G antibody to label neutrophils. The antibody was labeled with either AF647, MB488, or AF555. Images are displayed at identical window-level settings. Images were acquired sequentially with four lasers, except one laser was turned off in each row to determine the bleed-through signal. a, No 405 nm laser results in tumor cells being non-excited. b, No 663 nm laser with lack of Ly6G-AF647 excitation. c, No 559 nm laser with lack of Ly6G-AF555 excitation. d, No 473 nm laser with lack of Ly6G-MB488 excitation. AF: AlexaFluor. Scale bars: 50 µm.

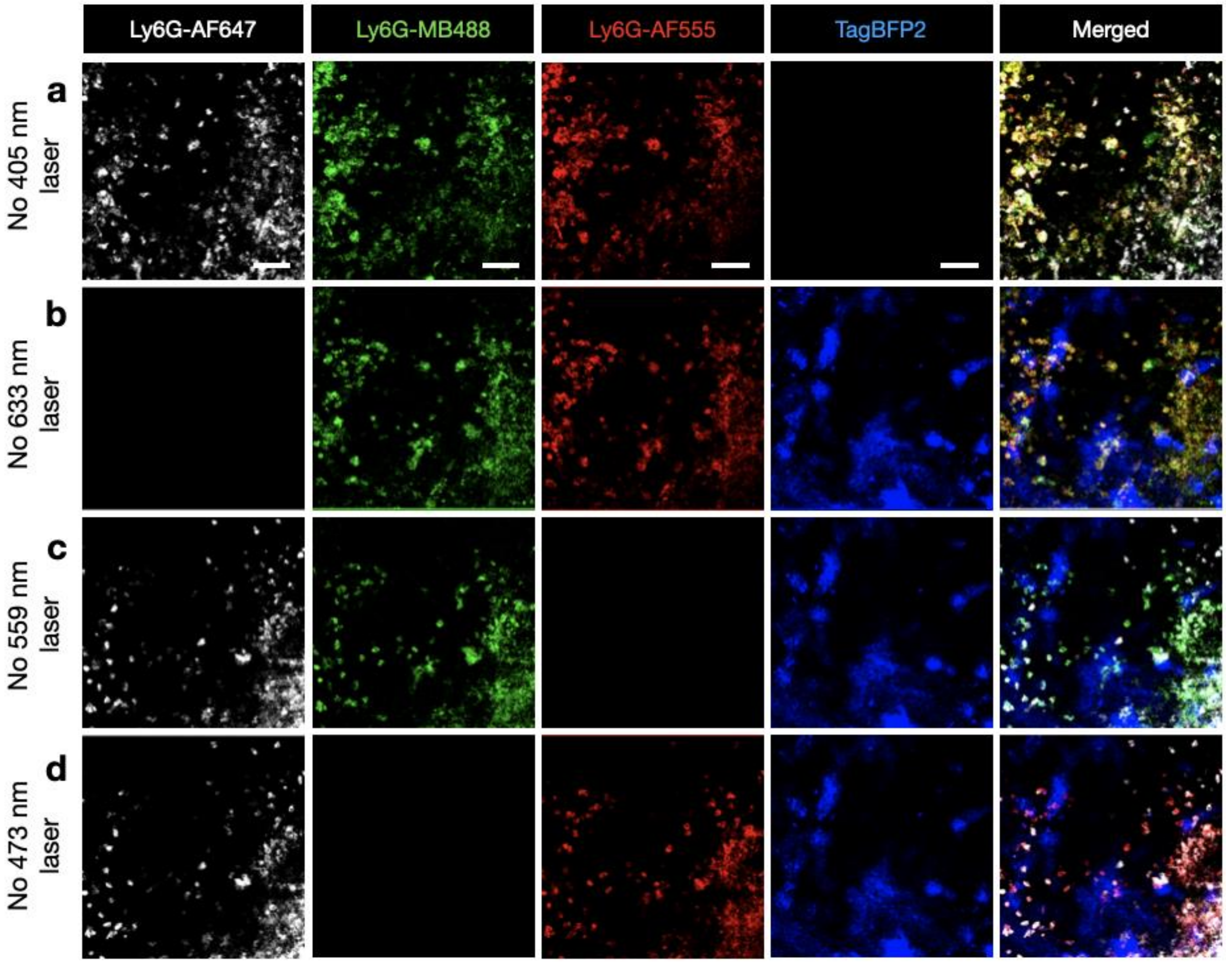

Fig. S8: Perfusion kinetics. The PWC is connected to dual syringe pumps, one to push liquid into the chamber and one to remove it. a, Low-resolution serial images of PWC with MC38 TagBFP2 cells in blue. The perfusate (dextran-rhodamine) is shown over time at the fastest 20 mL hr^{-1} flow rate. Note the rapid kinetics of perfusion throughout the entire chamber. The solution was switched to PBS at 0.9 mL elapsed for each rate, corresponding to 2.7 minutes for the 20 mL hr^{-1} rate, 3.6 min at 15 mL hr^{-1}, 5.4 min at 10 mL hr^{-1}, and 10.8 min at 5 mL hr^{-1}. For additional detail, see Movie S and 2. b, Chamber fluorescence graphed as a function of time for different pump settings showing perfusion kinetics. With the highest pump setting of 20 mL hr^{-1}, the PWC can be thoroughly perfused and cleared within five minutes. At the lowest perfusion rate tested (5 mL hr^{-1}), the entire cycle takes approx. 20 minutes.

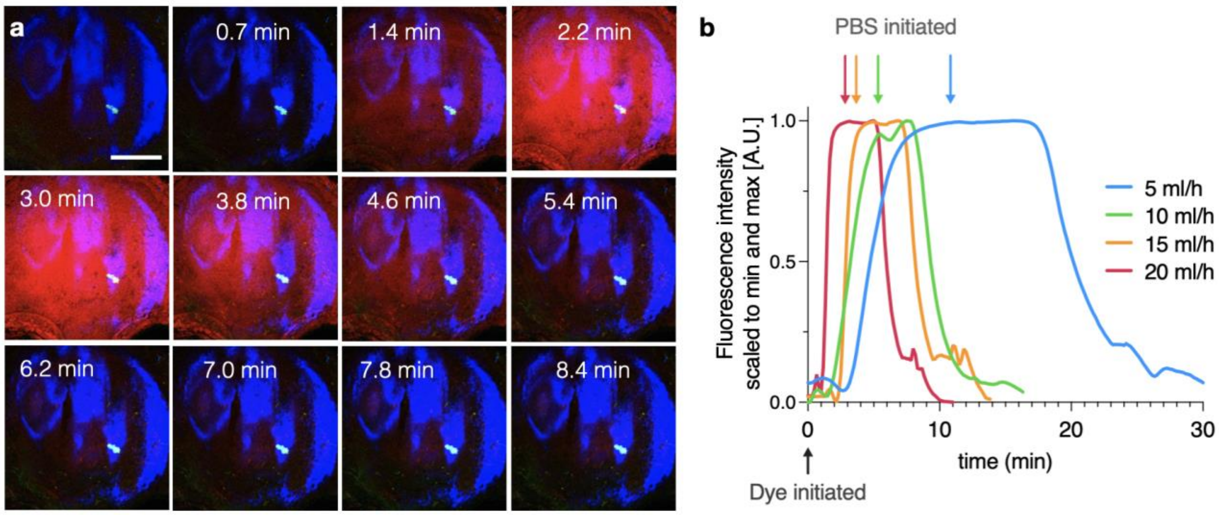

Fig. S9: Systemic vs. PWC antibody labeling. To determine whether antibodies perfused through the PWC behaved differently from standard workflows, we compared systemic and PWC-administered labeling. **a,** anti-Ly6G-AF647 administered systemically 24 hours before imaging identified neutrophils in the TME. **b-c,** The TME was perfused with two different anti-Ly6G antibodies labeled with SAFE-MB488 (green) and SAFE-AF555 (red). Note the good co-localization between the two PWC antibodies. **d,** There was very good co-localization between the systemically administered antibody from (a) and antibodies given by the perfusion window chamber in (b) and (c). **e,** SNR of cells in three different channels (n=3 experiments, mean shown). **f-h,** Pearson's correlation plots for co-localization of antibody pairs. Scale bar: 50 µm. TME: tumor microenvironment. SNR: signal-to-noise ratio.

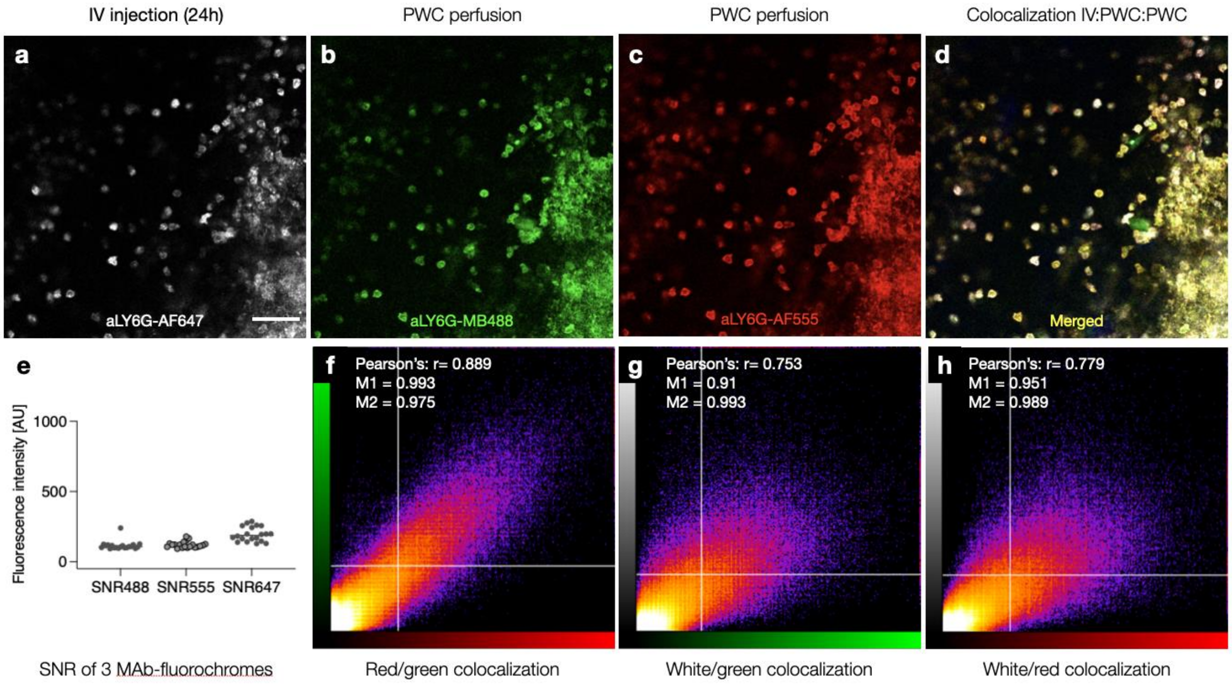

Fig. S10: Confocal depth imaging following IV or PWC staining of tumoral neutrophils with anti-Ly6G. a, anti-Ly6G-AF647 was administered systemically 24 hours before imaging and revealed neutrophils in the TME. **b-c,** The TME was perfused with two different anti-Ly6G antibodies labeled with MB488 (green) and AF555 (red). **d,** Merge channel. Note the good colocalization between IV- and PWC-administered antibody down to 84 µm. Blue: MC38-TagBFP2 is shown only in the merge channel. IV: intravenous. TME: tumor microenvironment. AF: AlexaFluor. Scale bar: 50 µm.

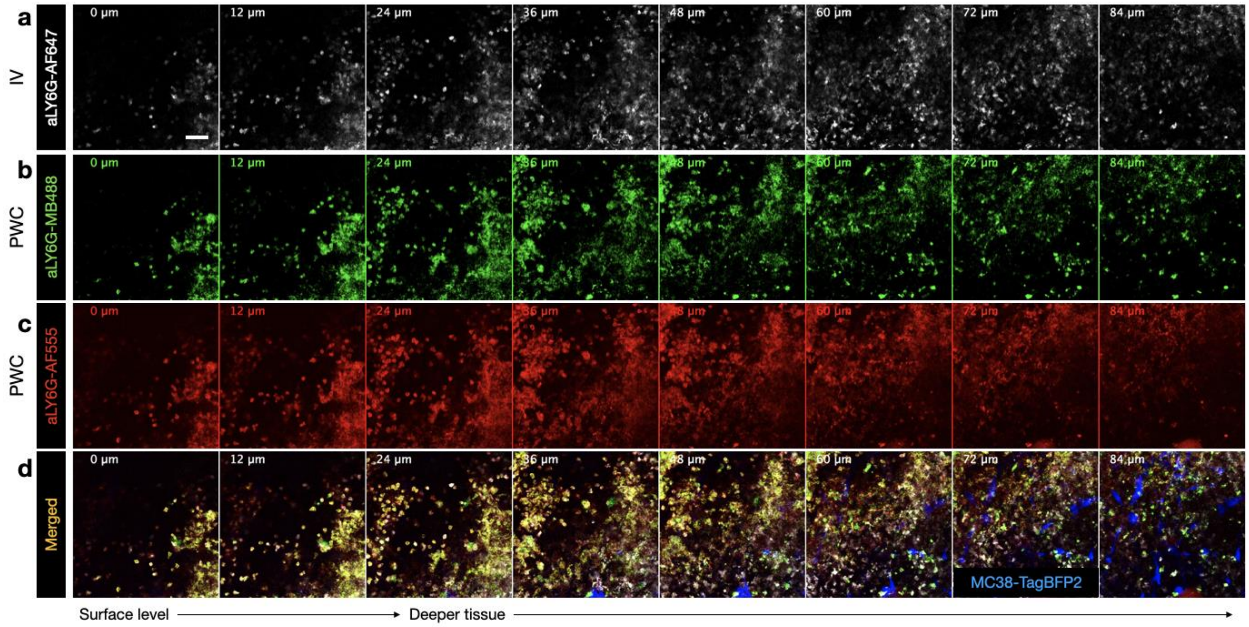

Fig. S11: In situ staining and cleaving. To determine whether serial staining could be performed in situ through the perfusion chamber, we tested a recently developed bioorthogonal method of staining followed by cleaving off antibody labels.[36] **a,** Pre-perfusion image showed no background. **b,** Chamber perfusion using an anti-Ly6G-AF647 antibody stained superficial neutrophils in part of the chamber. **c,** Following administration of the Hk-Tz scissors,[36] the signal returns to near background. **d,** Re-staining of the same section with a new anti-Ly6G antibody. **e,** Second round of cleaving. **f,** Quantification of fluorescent signal over time for the stain and cleave cycles for multiple cell ROI's. Since neutrophils are mobile, quantification was done on cohorts of cells rather than the same cell. n=3 experiments, one way ANOVA with Tukey multiple comparison test, $p<0.05$, with mean ±SD shown. ROI: region of interest. AU: arbitrary unit. Scale bar: 50 μm.

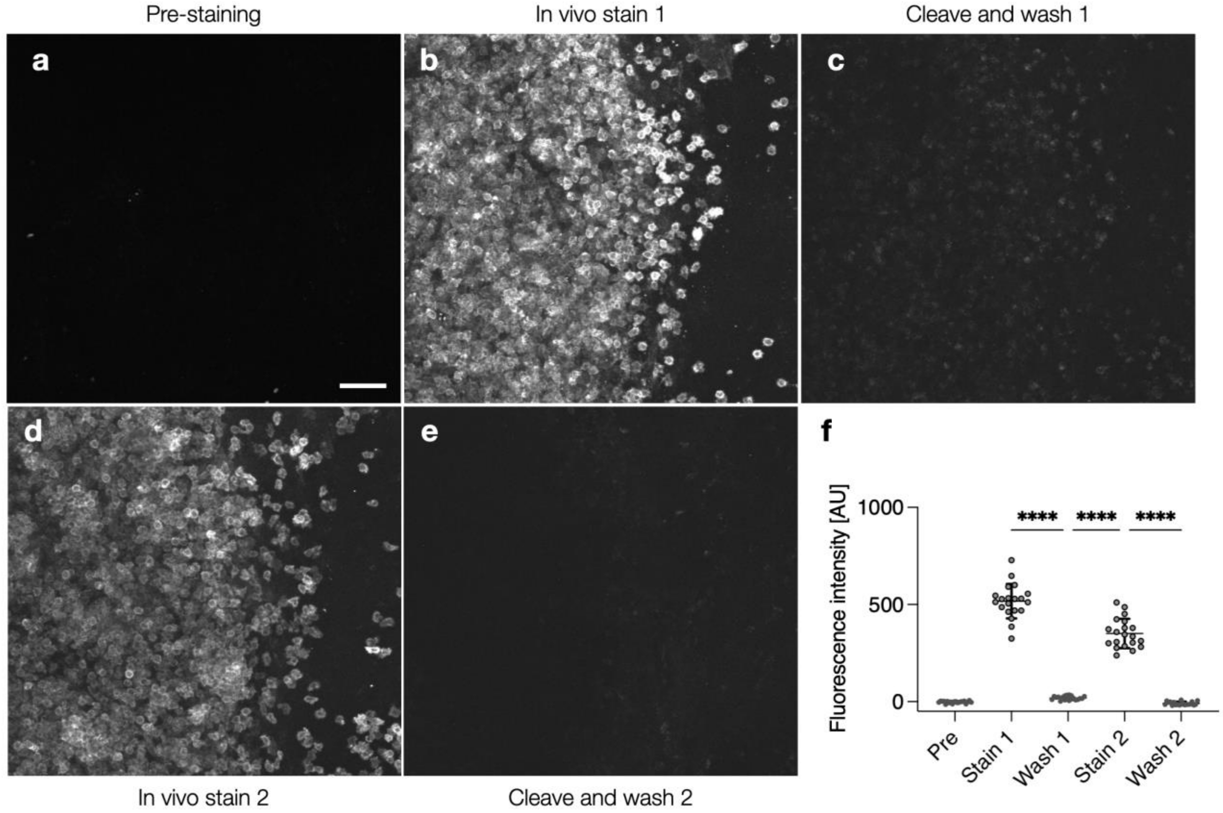

Fig. S12: Extracellular vesicle sampling from TME. To determine whether the tumor microenvironment could be safely sampled for biomarker analysis, PWC implanted with MC38 tumors were perfused with PBS to collect effluent. Samples were processed to isolate tissue-derived EV, then stained for CD9 and CD63, typical tetraspanin markers present in EV. **a,** Experimental setup for TME sampling. **b,** Sample images of EV from PWC effluent labeled with the pan-EV TFP (left), CD9-FITC (middle), and CD63-AF647 (right). Insets show negative controls (no EV, antibody only). Circled objects indicate EVs identified in the TFP channel (left) which co-labeled with the respective marker (middle, right). Small numbers of vesicles were identified within the TME, as expected. EV: extracellular vesicles. TME: tumor microenvironment. Scale bar: 500 nm.

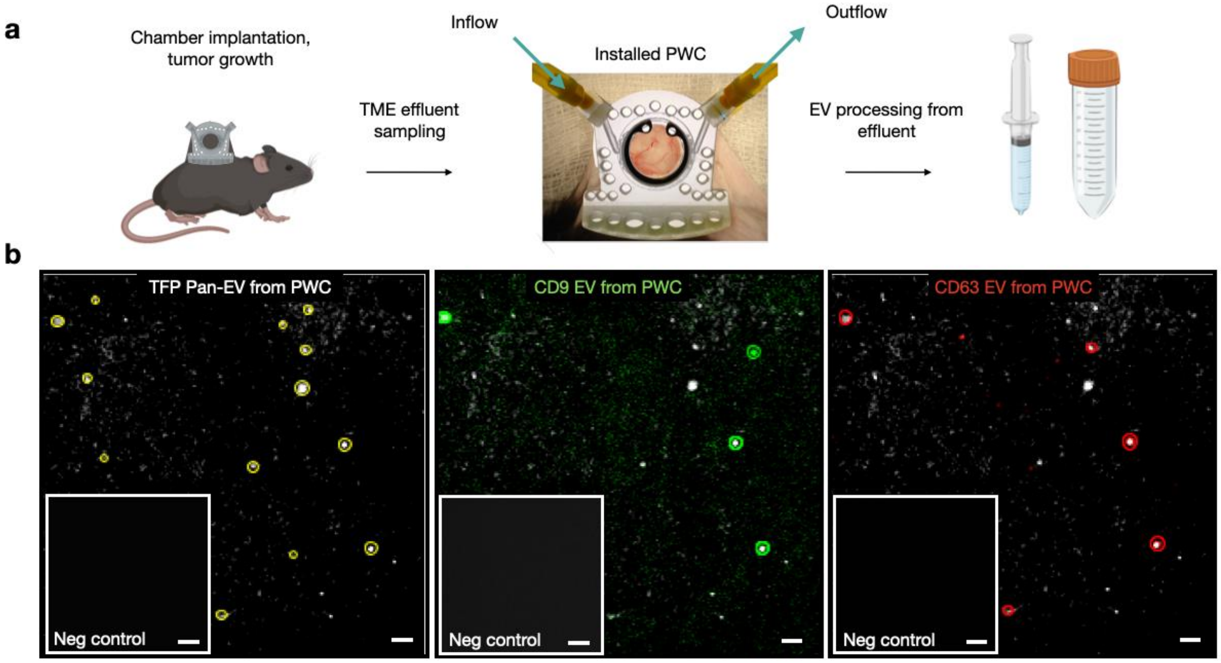

TABLES

Table S1: PWC engineering design factors that were explored to achieve the primary objective of stably transferring liquid in and out of the window chamber while keeping the system self-contained.

Factor	Constraints	Range of solutions tested	Outcome
**3D printing technology/material**	• Biocompatibility<br>• Resolution<br>• Price<br>• Minimizes weight<br>• Smooth surface<br>• MRI and CT compatible material<br>• Sterilizable	• Fused deposition / fused filament modeling<br>- Polystyrene-based (Stratasys printer)<br>- Stainless steel (MarkForged printer)<br>• Polyjet matrix<br>- Acrylic-based (Stratasys printer)	Polyjet matrix, transparent acrylic
**Wash protocol for channel unclogging**	• Must maintain part integrity<br>• Minimize duration	• NaOH and water washes<br>• Pressurized washes<br>• Freeze-drying	Freeze drying
**Connector type**	• Easy to swap<br>• Robust against pressure	• Full size Luer lock<br>• Mini luer<br>• Embedded mini luer<br>• Tube fitting, 18-22 gauge	Embedded mini luer
**Connector angle**	• Does not obstruct microscope objective	• 180˚ (flat)<br>• 90˚ (vertical)<br>• 45˚ (angled)	45˚
**Suture & screw hole size and distribution**	• Large enough for needlework<br>• Small enough to support spacers<br>• Spread to maintain tissue tautness	• 1.2 mm<br>• 1.5 mm<br>• 1.7 mm	1.5 and 1.7 mm hole diameter; side holes added
**Lip height for tension ring**	• Does not obstruct microscope objective<br>• Stably supports ring placement	• 1 mm<br>• 1.5 mm<br>• 2 mm	1.5 mm
**Backing thickness**	• Minimize weight<br>• Minimize overall construct thickness when implanted<br>• Maintain integrity	• 1 mm<br>• 1.5 mm<br>• 2 mm	1.5 mm
**Backing angle**	• Minimizes pressure on body<br>• Keeps chamber straight	• 20˚<br>• 35˚<br>• 40˚<br>• 45˚	35˚

Factor	Constraints	Range of solutions tested	Outcome
**Coverglass opening size**	• Supports coverglass easy removal<br>• Minimizes gap and space for leakage (i.e., must be snug)	• 12 mm<br>• 12.1 mm<br>• 12.2 mm<br>• 12.3 mm<br>• 12.4 mm<br>• 12.5 mm<br>• 13 mm<br>• 13.5 mm	12.4 mm (for 12 mm coverglass)
**Edge length for coverglass**	• Supports coverglass and tension ring<br>• Minimizes leakage<br>• Maximizes imaging area	• 1.25 mm<br>• 1.5 mm<br>• 2 mm<br>• 2.5 mm	1.25 mm
**Microfluidic manifold design**	• Can be robustly unclogged<br>• Even & gentle perfusion<br>• Preserves overall part integrity	• Single tier<br>• Short vs long channels<br>• Oval vs rectangular spread<br>• Bifurcation, 2-tier<br>• Bifurcation, 3-tier<br>• Sharp vs rounded angle changes	Rectangular spread, 3-tier bifurcation, short channels, rounded angles
**Channel number**	• Minimal clogging<br>• Even perfusion	• 4 channels<br>• 5 channels<br>• 6 channels<br>• 8 channels	8 channels
**Inlet size**	• Even perfusion<br>• Stable at connection port	• Constant vs tapered diameter<br>• 0.4 mm<br>• 0.6 mm<br>• 0.9 mm	0.9 mm constant diameter
**Channel size and wall thickness**	• Minimize working distance for objective<br>• Integrity of fabrication	0.4 mm diameter; minimum resolution for printing	0.4 mm minimum feature diameter and wall thickness
**Skin-facing seal type**	• Prevents liquid leakage from chamber on skin-side<br>• Minimizes swelling and suppuration	• No raised seal<br>• PDMS gasket<br>• Raised solid rim recessed from opening<br>- 0.5 mm height<br>- 1 mm<br>- 1.5 mm	1.6 mm wide, 0.5 mm height, recessed by 0.75 mm

Factor	Constraints	Range of solutions tested	Outcome
**Coverglass-facing seal type**	• Removable<br>• Non-obstructive<br>• Leak-proof	• Cyanoacrylate glue<br>• Waterproof medical tape<br>• Liquid bandage<br>• Plastomer thread sealant tape<br>• Mounting putty<br>• Silicone sealing gum	Cyanoacrylate glue
**Installation type**	• Preserve tissue integrity<br>• Support tumor growth	• Permanent<br>• Temporary	Permanent installation

Table S2: PWC components.

Item	Product ID	Vendor
3D printed PWC, backing, plugs	PJM J850 3D printing VeroClear	R&D Technologies, Stratasys J850 printer
Microfluidic connectors, male mini luer	CS-10000095	Darwin microfluidics, ChipShop
Teflon (PTFE) tubing, 22G	Cat.# BB311-22-100′ Roll	Scientific Commodities Inc
Silicone tubing, 0.8 mm (1/32") ID / 4.0 mm OD / 1.6 mm (1/16”)	SHE-TUB-SIL-0.5*0.8	Darwin Microfluidics
3-way stopcock	MX9311L	Smiths Medical
Syringe pumps	Programmable Syringe Pump BS-8000	Braintree Scientific
Stainless Steel Dispensing Needle with Luer Lock Connection, 1/2" Needle Length, 23 Gauge	75165a684	McMaster-Carr
Coverglass, 12 mm	72231-01	Electron Microscopy Sciences
Cyanoacrylate glue	Instant-Bond Adhesive Loctite® 406	McMaster-Carr
Tension ring/Internal Retaining Rings	91580A146<br>29/64” ID	McMaster-Carr
spacers	Threaded small aluminum spacers, Model: SP100-TH	APJ Trading Co., Inc.
Screws and nuts	0-80 thread size nuts (91841A115) and screws 3/8” length (91772A057)	McMasterCarr

Item	Product ID	Vendor
DPBS	14190144	Thermofisher Scientific

Table S3: Antibodies and stains. Antibodies were diluted to 2-4 µg/mL for imaging applications unless otherwise specified.

Target protein	Target population	Product	Clone	Vendor	Catalog #	Fluorophores
MHCII	Antigen-presenting cells	Purified anti-mouse I-A/I-E Antibody	M5/11 4.15.2	BioLegend	107602	SAFE conjugated MB488, AF555, AF647
MHCI	Antigen-presenting cells	MAb anti-mouse MHC Class I (H-2)	M1/42.3.9.8	BioXcel	BE0077	SAFE conjugated MB488, AF555, AF647
CD11b	Monocytes, granulocytes, NK cells	InVivoMAb anti-mouse/human CD11b	M1/70	BioXcel	BE0007	SAFE conjugated MB488, AF555
CD11c	Dendritic cells	Purified anti-mouse CD11c Antibody	N418	BioLegend	117302	SAFE conjugated MB488, AF555, AF647
CD4	T helper cells	Purified anti-mouse CD4 Antibody	GK1.5	BioLegend	100402	SAFE conjugated MB488, AF555, AF647
CD8	T cytotoxic cells	MAb anti-mouse CD8α	53.6.7	Bio X cell	BE0004-1	SAFE conjugated MB488, AF555, AF647
F4/80	resident macrophage	Purified anti-mouse F4/80 Recombinant Antibody	QA17A29	BioLegend	157302	SAFE conjugated MB488, AF555, AF647
CD68	Macrophage	Purified anti-mouse CD68 Antibody	FA-11	BioLegend	137001	SAFE conjugated MB488, AF555, AF647
CD19	B cells	Purified anti-mouse CD19 Antibody	1D3/CD19	BioLegend	152402	SAFE conjugated MB488, AF555, AF647
CD45	Immune cells broadly	InVivo MAb anti-mouse CD45.2	45.2	Bio X cell	BE0300	SAFE conjugated MB488, AF555, AF647
Ly6G	Neutrophils	MAb anti-mouse Ly6G	1A8	Bio X cell	BE0075-1	SAFE conjugated MB488, AF555, AF647
Ly6G	Neutrophils	Alexa Fluor 647 anti-mouse Ly6G	1A8	BioLegend	127610	Commercially conjugated with AF647

FCR	Antibody Fc gamma receptors RII RIII	anti-mouse CD16/CD32 receptors Fc gamma receptors II and III	2.4G2	Bio X cell	BE0307	N/A; used as blocking agent 100 µg injected by IV, 25 µg/mL in staining solution
Hoechst	Nuclei	Intercalating dye	N/A	Invitrogen	H3570	Hoechst33342
Dextran-FITC (MW= 2,000 kDa)	Fluid flow in vascular space	Fluorescein isothiocyanate -dextran	N/A	Sigma-Aldrich	FD2000S	FITC
Dextran-Rhodamine (MW= 2,000 kDa)	Fluid flow in vascular space	Dextran, Rhodamine B/RITC labeled	N/A	Nanocs Inc.	DX2000-RB-1	Rhodamine
Lectin-Rhodamine	Vasculature	Griffonia (Bandeiraea) Simplicifolia Lectin I (GSL I, BSL I), Rhodamine	N/A	Vector Labs	RL-1102-2	Rhodamine
CD9	EV marker	FITC anti-mouse CD9 antibody	MZ3	BioLegend	124807	FITC
CD63	EV marker	AlexaFluor 647 anti-mouse CD63 antibody	NVG-2	BioLegend	143921	AF647

Table S4: Cell lines.

Cell Line	Reporter	Cell type/model	Primary use	Manufacturer / obtained from	Catalog Number
MC38 H2Bapple	H2Bapple	Murine colorectal carcinoma	PWC implantation	CSB generated from original MC38 cell line obtained from Mark Smyth	RRID:CVCL_B288
MC38 H2BGFP	H2B-GFP	Murine colorectal carcinoma	PWC implantation	CSB generated from original MC38 cell line obtained from Mark Smyth	RRID:CVCL_B288
MC38 mTag BFP2	mTag-BFP	Murine colorectal carcinoma	PWC implantation	CSB generated from original MC38 cell line obtained from Mark Smyth	RRID:CVCL_B288
HT1080	FUCCI	Human fibrosarcoma	PWC implantation	CSB generated from original ATCC cell line	RRID:CVCL_0317
B16-F10	H2B-apple	Murine, skin melanoma	PWC implantation	ATCC	CRL-6475
WH6042 KPT ($Kras^{LSL\text{-}G12D}$ $Trp53^{Fl/Fl}$ $Rosa26^{LSL\text{-}tdTomato}$)	tdTomato	Murine pancreatic ductal adenocarcinoma	*In vitro* assay, PWC implantation	CSB, kind gift from William Hwang; Harvard	N/A
IMR90	N/A	Human normal lung fibroblast	*In vitro* assay	ATCC	CCL-186

Table S5. Mouse strains.

Name	Manufacturer	Catalog Number	N
C57BL/6J	Jackson Laboratory	Stock# 000664<br>RRID:IMSR_JAX:000664	n = 49
merTK-GFP	>15 generation of backcrosses from Jax NOD.Cg-*Mertk*^{em1Doi} *Prkdc*^{scid}/Rwei into C57BL/6J	NOD.Cg-*Mertk*^{em1Doi} *Prkdc*^{scid}/Rwei<br>Strain #:036071<br>RRID:IMSR_JAX:036071	n = 9
IL12-eYFP	Jackson Laboratory	Strain # 015864<br>RRID:IMSR_JAX:015864	n=5
